# Exploring Light Stability and Trapping Mechanisms in Organic Thin-Film Transistors for High-Brightness MicroLED Integration

**DOI:** 10.3390/ma17225643

**Published:** 2024-11-19

**Authors:** Chia-Hung Tsai, Yang-En Wu, Chuan-Wei Kuo, Ting-Chang Chang, Li-Yin Chen, Fang-Chung Chen, Hao-Chung Kuo

**Affiliations:** 1 Department of Photonics, College of Electrical and Computer Engineering, National Yang Ming Chiao Tung University, Hsinchu 30010, Taiwan; sppsai.ee12@nycu.edu.tw (C.-H.T.); ivanwu.ee12@nycu.edu.tw (Y.-E.W.); lychen@nycu.edu.tw (L.-Y.C.); 2 Smartkem Ltd., Manchester M9 8GQ, UK; 3 Department of Materials and Optoelectronic, National Sun Yat-sen University, Kaohsiung 80424, Taiwan; bike615353@gmail.com; 4 Department of Physics, National SunYat-sen University, Kaohsiung 80424, Taiwan; tcchang3708@gmail.com; 5 Center of Crystal Research, National Sun Yat-sen University, Kaohsiung 80424, Taiwan; 6 Center for Emergent Functional Matter Science, National Yang Ming Chiao Tung University, Hsinchu 30010, Taiwan

**Keywords:** organic thin-film transistors (OTFTs), monolithic MicroLED integration, charge-trapping mechanisms, light-induced trapping, suppression of photo-induced charges

## Abstract

Organic thin-film transistors (OTFTs), benefiting from a low-temperature process (≤120 °C), offer a promising approach for the monolithic integration of MicroLED structures through organic-last integration. Previous research has demonstrated that small-molecule/polymer binder-based organic semiconductor deposition, utilizing the vertical phase separation mechanism, can achieve good device uniformity while preserving high field-effect carrier mobility. However, the stability of OTFTs under light exposure at the device level remains underexplored. This study investigates the effects of various light irradiation conditions on OTFTs and delves into the underlying mechanisms of the light-trapping effect. Based on these findings, we propose an optimal OTFT design tailored for driving MicroLED displays at high operational brightness, ensuring both performance and stability.

## 1. Introduction

**Figure 1:**
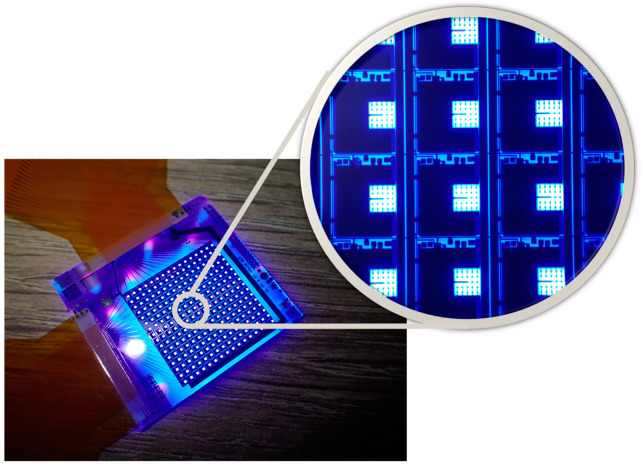
Monolithic MicroLED driven by OTFTs.

Organic thin-film transistors (OTFTs) have emerged as a promising candidate for flexible electronics [1], wearable devices, and display technologies due to their advantages in low-temperature processing, mechanical flexibility [2], and compatibility with large-area fabrication techniques [1,3,4,5,6]. One of the most intriguing applications of OTFTs lies in their potential for seamless integration with MicroLED arrays [7], which are known for their exceptional brightness, contrast, and energy conversion efficiency. MicroLED displays have recently become a popular topic in the display industry [8,9,10,11]. Monolithic integration of OTFTs and MicroLEDs can significantly streamline the manufacturing process and reduce production costs, making it an attractive option for next-generation displays, as demonstrated in Figure 1. However, despite these advantages, the issue of OTFTs’ reliability still needs to be addressed [12,13,14], especially when exposed to high-brightness environments. Light-induced degradation, including the formation of photo-induced trapped charge carriers and the resultant shifts in device characteristics, poses a serious obstacle to the reliable operation of OTFTs in display technologies [15,16,17].

Past studies have demonstrated that organic semiconductors, which form the active layer in OTFTs, can achieve excellent charge mobility in a region of 0.1 cm^2^/V·s to 12.3 cm^2^/V·s [18], as well as device uniformity through vertical phase separation mechanisms [19] involving small molecules and polymer binders [12,20]. This has enabled the development of OTFTs with performance characteristics that are suitable for integration with MicroLED arrays. However, the behavior of these devices under continuous light exposure has not been thoroughly investigated, especially in scenarios where the devices must operate at the high luminance levels required for modern display applications. Under such conditions, the organic semiconductors in OTFTs are susceptible to photo-induced trapping [21,22], a phenomenon where excitons generated by incident light dissociate into free carriers, which then become trapped within the device. These trapped carriers can cause long-lasting changes in the electrical properties of the OTFTs, leading to instability in the threshold voltage (Vth) and hysteresis in the device’s transfer characteristics (drain current–gate voltage, ID-VG).

The focus of this study is to systematically explore the effects of light exposure on OTFTs, particularly under conditions simulating the operational environment of high-brightness MicroLED displays. By examining the trapping mechanisms that occur under illumination conditions, we aim to identify the specific factors that contribute to the degradation of OTFTs’ performance. In doing so, we propose transistor designs based on a light-blocking layer to mitigate these effects. This light-blocking layer is intended to shield the active organic semiconductor regions from incident light, thereby reducing the likelihood of photo-induced trapping and improving the overall stability of the device.

Through a combination of experimental results and device energy band analysis, we seek to provide a deeper understanding of the light-stability challenges faced by OTFTs and offer practical insights into their design optimization, in order to ensure that OTFTs can reliably drive MicroLED displays under high-brightness operational conditions, without suffering from performance degradation due to prolonged exposure to light. By addressing these critical issues, this research contributes to the advancement of organic electronics, particularly in applications that demand high brightness and long-term stability, such as high-contrast display technologies and wearable devices. This work highlights the importance of considering light stability in OTFT design but also lays the groundwork for future developments in the field of organic semiconductor devices tailored for display applications.

## 2. Experiment and Fabrication Process

### 2.1. Monolithic MicroLED Integration

**Figure 2:**
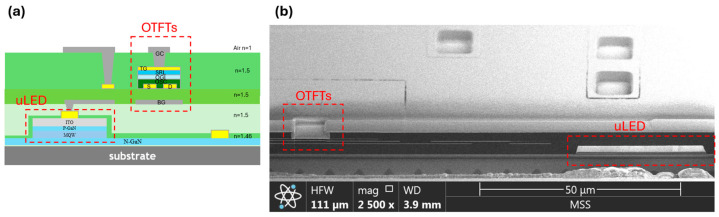
Cross-sectional image of monolithic MicroLED: (**a**) relative position of OTFTs and MicroLED, and (**b**) FIB image.

Figure 2 depicts the proposed organic-last integration architecture [7], where the OTFT active matrix (AM) array is directly fabricated on top of the MicroLED array. The MicroLED array is constructed on a 4-inch sapphire substrate through metalorganic chemical vapor deposition (MOCVD) processes, which include layers such as n-doped GaN (N-GaN), a multi-quantum well (MQW), p-doped GaN (P-GaN), and an ITO electrode. The OTFT adopts a 5-mask top-gate bottom-contact (TGBC) structure, incorporating metal shielding layers. Detailed steps of the process are illustrated in Section 2.2. For the AM array’s creation, a thick micrometer-scale metal interconnect layer is added to the MicroLED plane to provide power (VDD) and ground (VSS) lines, which are essential for managing large current loads. However, the substantial topography of the thick metal layer poses a risk of electrical shorts at the crossover points between power and data interconnects. To mitigate this, a thick polymer planarization layer is applied over the MicroLED plane before metal deposition for both data interconnects and OTFT bottom shielding. A buffer layer is subsequently added, offering a surface that is conducive to uniform OSC channel crystallization, and forming a high-quality back-channel interface.

To connect the MicroLED contact to the OTFT source and drain (S&D), a post-OTFT via process is implemented, in which the OTFT-to-MicroLED via is formed after the OTFT is completed. This method preserves the planar buffer layer surface, free from ion charge trapping, enabling an optimal OSC phase separation process and consistent electrical properties. After the OTFT is fabricated, the passivation layer (PV) is deposited over all device areas. Throughout the integration process, the planarization, buffer, organic semiconductor (OSC), gate insulator (OGI), stress-release layer (SRL), and PV are all applied using spin-coating. Metal electrodes and interconnects are formed by sputtering and patterned via photolithography and wet etching. Via formation and OSC patterning are performed using dry etching. All of these steps are compatible with the existing III-V MicroLED manufacturing infrastructure. The organic-last integration processes, conducted at low temperatures (<150 °C), are thus fully compatible with MicroLED fabrication, enabling direct construction of the AM array without compromising MicroLED performance.

### 2.2. Organic TFTs’ Process and Fabrication

**Figure 3:**
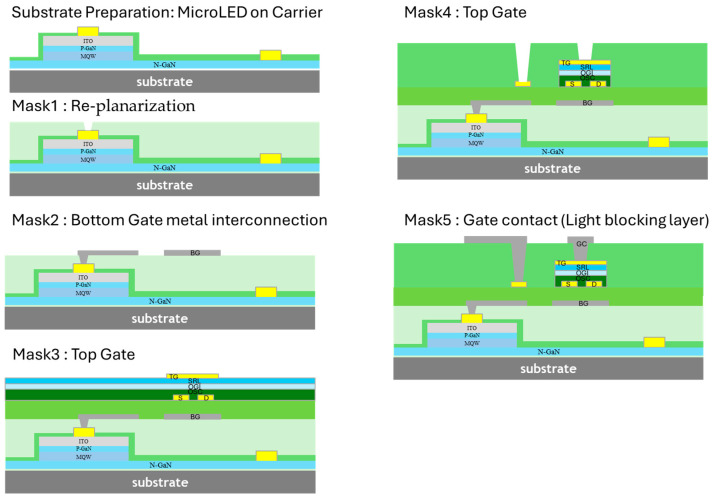
Five-mask organic TFTs’ process flow.

As shown in Figure 3, after the thick Au metallization was completed, the substrate was re-planarized with a spin-coated layer of TRUFLEX™ organic planarization, a UV-crosslinkable acrylate polymer. The planarization was cured for 2 min under broadband UV light (i, g, and h lines) in ambient conditions, with an energy dose of 4200 mJ/cm^2^. To enhance the surface energy and improve the adhesion of the planarization layer to the substrate’s uneven topography, a light O_2_ plasma treatment was applied. Additionally, a thiol-based adhesion promoter was spin-coated in IPA to facilitate stronger bonding between the planarizer and Au regions. Following crosslinking, the wafer was baked for 60 min at 150 °C.

Vias to the Au contact pads were formed by first spin-coating a positive-tone resist onto the planarizer, which was then soft-baked for 1 min on a hotplate. The resist was patterned using a Heidelberg MLA direct lithography system with a 405 nm exposure wavelength, and subsequently developed in a 2.38% TMAH aqueous solution to create the resist mask for the vias. The vias were etched using reactive ion etching (RIE) with O_2_ plasma for 7.5 min under 200 millitorr pressure in an Oxford Plasma Lab 800+. After etching, the resist was flood-exposed to UV and removed in TMAH developer.

Next, a Mo/Al/Mo tri-layer was deposited via sputtering to form the back-gate layer, which was patterned through photolithography and wet-etched using a phosphoric/acetic/nitric acid solution. This layer served as both the transistor’s back-gate electrode and a light-blocking layer to prevent unwanted light-induced doping of the OSC. Subsequently, a TRUFLEX™-based dielectric layer was spin-coated and cured similarly to the planarization layer.

Following this, 50 nm thick Au S&D electrodes were sputtered, patterned using photolithography, and etched with a KI/I_2_ aqueous solution. Once the S&D electrodes were defined, a light plasma treatment was used to enhance the surface energy of the base dielectric. Then, a self-assembled monolayer (SAM) and an OSC were spin-coated, followed by brief baking at 100 °C for 1 min. The OGI layers, along with an acrylate-based sputter-resistant layer (SRL), were subsequently applied. The gate metal (GM), consisting of a 50 nm layer of Au, was applied via sputtering and patterned through photolithography. After the resist was removed using a flood exposure and development process, RIE with a 100 sccm/20 sccm O_2_/Ar plasma was employed to selectively etch away the OSC, OGI, and SRL layers that were not shielded by the GM. The etching process extended into the base layer but was carefully controlled to avoid penetrating the underlying back-metal layer.

Subsequently, the PV (TRUFLEX™) was deposited using spin-coating, followed by an initial baking at 115 °C for 1 min. This was crosslinked under UV light (4200 mJ/cm^2^) for 2 min, after which the sample underwent a secondary baking at 120 °C for an additional 5 min. Via holes were patterned in a manner similar to the planarization layer, with etching proceeding until the back-gate layer was exposed. These vias established connections between the back-gate, S&D, GM, and interconnect layers. The final step involved sputtering a Mo/Al/Mo tri-layer interconnect layer, referred to as a gate contact (GC), which was then patterned and etched using photolithography and wet etching to complete the fabrication process.

### 2.3. Methods and Test Equipment

In this work, the electrical characteristics were analyzed using an Agilent B1500A semiconductor device analyzer by Agilent Technologies. Agilent Technologies is headquartered in Santa Clara, CA, USA, and the analyzer was manufactured in Hachioj, Japan, a Cascade Microtech M150 measurement platform, manufactured by FormFactor, based in Livermore, CA, USA, and a LakeShore CPX-VF/TTP6 measurement platform, produced by Lakeshore Semiconductor located in Kingsford, MI, USA, were used. While a Hitachi U-3900H spectrophotometer was used to measure the transmission spectra, made by Hitachi Energy in Tokyo, Japan.

## 3. Results and Discussion

In the recent OTFT literature, research has largely focused on the application of OTFTs for gas or biosensors [23,24] and on evaluating their reliability under challenging operating conditions, such as self-heating stress (SHS) and hot-carrier stress (HCS) [6,25,26,27]. In parallel, studies on a-IGZO TFTs, which also have an active layer that is highly sensitive to light interference, underscore the importance of addressing this sensitivity. A common approach to managing light interference in a-IGZO TFTs involves using a light-shielding layer to protect the active region. Standard methods include adding this layer beneath the transistor, where it also functions as the bottom gate, or expanding the top-gate area to provide more coverage [28,29]. However, these configurations increase the device’s CGD parasitic capacitance, resulting in RC delay, which can impact the device’s performance. An alternative method is to position the light-shielding layer above the transistor [30], although this approach necessitates an additional photomask, thereby increasing the fabrication costs. In response to these limitations, this study proposes a new approach that involves placing the light-shielding layer on the GC, away from the transistor itself, effectively preventing any increase in CGD. Furthermore, by connecting the GC to the gate wiring in the circuit design, this configuration eliminates the need for additional photomasks, leading to reduced processing costs and a more efficient fabrication process.

### 3.1. Experiment Flow and OTFTs’ Device Characteristics

**Figure 4:**
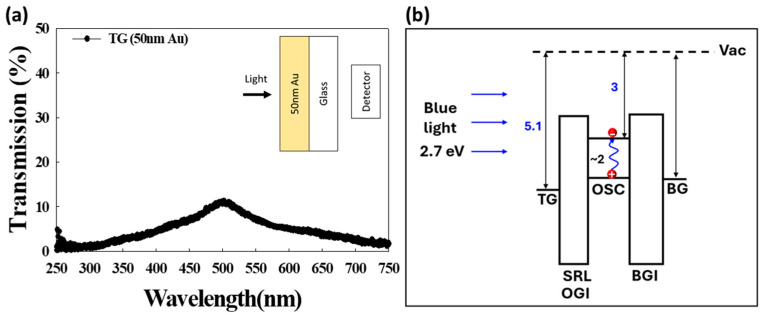
(**a**) The transmission spectra of the TG metal structured with a 50 nm Au layer. The thin layer of TG metal is semi-transparent in light transmission across the visible spectrum, particularly between 400 nm and 700 nm. (**b**) A schematic of the device structure, indicating the path of blue light with an energy range of 2.7 eV as it penetrates the TG layer and reaches the organic semiconductor (OSC) layer, where exciton generation and dissociation can occur.

In Figure 4a, the transmission spectra of the TG (top gate) structure, which includes a 50 nm Au (gold) layer, are shown. The black curve represents the transmission under ultraviolet/visible spectrophotometer measurement. This thin TG metal layer demonstrates partial transparency in the visible spectrum, with particularly notable transmission in the 400 to 700 nm wavelength range. This semi-transparency allows certain wavelengths of light, including blue light, to penetrate through the TG layer, making it possible for the light to reach underlying layers, which is essential for further optical interactions in the device structure. The OSC is a typical p-type semiconductor with a bandgap of approximately 2 eV [31], as shown in Figure 4b, while the accompanying insulating layers are made from wide-bandgap materials. The blue light is absorbed by the OSC layer, influencing the energy band configuration, as shown in the right-hand diagram. This absorption leads to exciton generation and dissociation, further impacting the device’s electrical characteristics under illumination.

**Figure 5:**
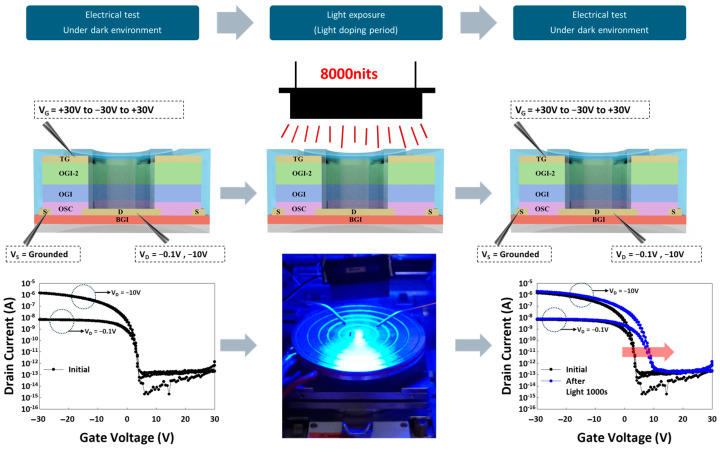
OTFT device under varying conditions of light exposure. The test process sequence consists of three steps, beginning with an initial transfer characteristic (ID-VG) electrical test conducted in a dark environment to establish the baseline performance characteristics of the device. The middle panel illustrates the device being exposed to blue light with an intensity of 8000 nits for a light-doping period. Following the light exposure, the device was again tested in a dark environment to assess any changes in its electrical properties post-illumination.

Figure 5 illustrates the experimental setup designed to investigate trapped charges generated purely by illumination, without any external electric field applied. The process begins with an initial electrical test under dark conditions to establish the baseline characteristics of the OTFT device. Next, the device is exposed to blue light at 8000 nits during a light-doping period, which leads to the generation of trapped charges within the OSC layer. These trapped charges are created solely due to the incident light, independent of any applied electric field. To evaluate whether these trapped charges possess long lifetimes, the device is subjected to a subsequent electrical test in the dark to monitor any persistent changes in its behavior.

The shift in the electrical characteristics, highlighted by the red arrow in the final graph, suggests that the trapped charges generated by light exposure are indeed long-lived, as the device’s behavior remains altered even after returning to dark conditions. This long-lifetime trapping significantly affects the device’s performance, particularly in environments where prolonged light exposure is present.

### 3.2. Discussion of Photo-Induced Charge-Trapping Mechanism

**Figure 6:**
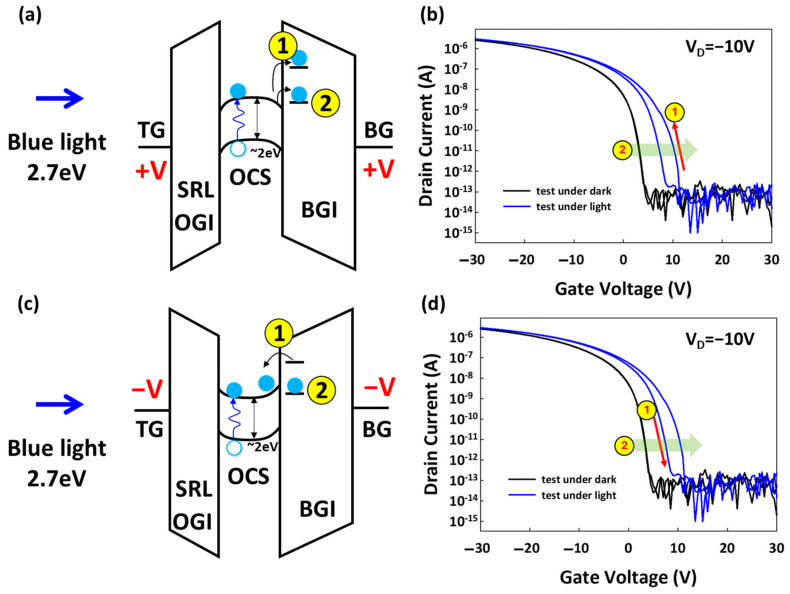
(**a**) Energy band diagram during light exposure with a positive gate voltage (VG) applied. (**b**) ID-VG curve, indicating VG forward sweep (+30 V to −30 V). (**c**) Energy band diagram during light exposure with a negative VG applied. (**d**) ID-VG curve, indicating VG reverse sweep (−30 V to +30 V).

Figure 6 presents the operational behavior of organic thin-film transistors (OTFTs) under darkness and blue-light illumination, where the measurements were conducted while the gate voltage (VG) was swept from +30 V to −30 V to +30 V. Both the bottom gate (BG) and top gate (TG) were held at the same potential, ensuring equal electrostatic control.

In the absence of blue-light illumination, the organic thin-film transistors (OTFTs) exhibit distinct electrical behavior compared to when illuminated. The measurements were again conducted with the VG sweeping from +30 V to −30 V to +30 V under dark conditions. Without light, the hysteresis window between the forward and reverse voltage sweeps was significantly reduced. This indicates that the photogenerated charge carriers, which contribute to the trapping and de-trapping phenomena under illumination, were absent. The hysteresis observed in the dark state was negligible. The Vth shift was observed in the illumination period rather than under darkness. The neglected Vth shift suggests that fewer trapped charges were accumulated in the dark.

**Mechanism 1:** Hysteresis. During the voltage sweep from positive to negative (VG = +30 V to VG = −30 V), a hysteresis effect was observed, characterized by a distinct window between the forward and reverse sweeps of the ID-VG curve. This hysteresis suggests the occurrence of charge trapping and de-trapping phenomena during the sweep. The window effect (labeled as Mechanism 1) indicates a lag between the charge injection and extraction processes, which is influenced by the presence of trap states within the semiconductor.

- Shallow traps (1): These are energy states located close to the conduction or valence band edges, leading to minor Vth shifts as these charges are easily captured and released.

**Mechanism 2:** Vth Shift. Additionally, a Vth shift was observed during the sweep. This Vth shift is indicative of trapped charges accumulating in the device. Based on energy band theory, these trapped charges can be attributed to two types of defects:

- Deep traps (2): These are energy states situated deeper within the bandgap, causing more significant Vth shifts, as these charges are more likely to remain trapped for longer periods.

The combined effects of these shallow and deep traps explain the hysteresis behavior and Vth shift observed under continuous illumination, providing insight into the charge-trapping mechanisms in OTFTs under operational conditions.

### 3.3. Optimization of TFT Design to Suppress Light-Induced Degradation

**Figure 7:**
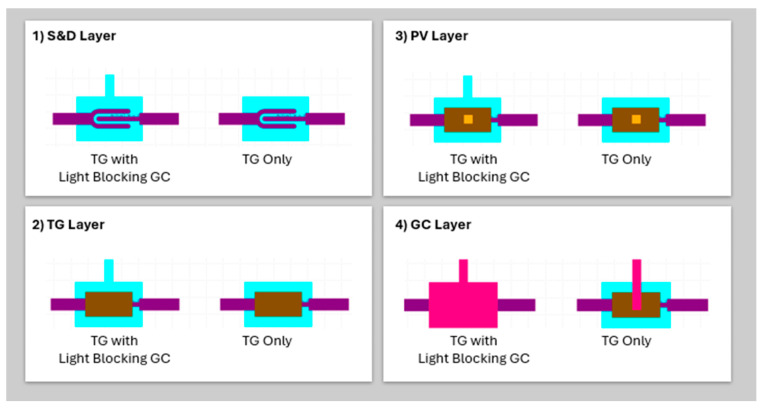
The structural differences between the original top gate (TG) design and the modified TG design with the inclusion of a light-blocking layer. These comparisons are made across four critical layers of the OTFT.

Due to the non-collimated nature of light, phenomena such as internal reflection, refraction, and scattering can occur within the device. The process flow and design optimization are shown in Figure 7. In the TG-only structure, the effects of light make it difficult to effectively block light from reaching the active layers. As a result, the TG-only configuration proves inadequate in preventing light-induced phenomena such as charge trapping. To address this issue, an additional GC layer is introduced, which functions as a light-blocking layer. This extra layer enhances the ability to shield the active regions from unwanted light exposure, thereby minimizing the detrimental effects of light on the device’s performance. The GC layer plays a crucial role in distinguishing between the two device designs: the original TG configuration, and the modified TG with light-blocking layer design. While the S&D, PV, and TG layers remain identical in both designs, the GC layer differs significantly, as it is responsible for incorporating the light-blocking feature.

In the TG-only design, the GC layer is relatively straightforward, serving the primary function of electrically connecting the TG to the external circuitry. However, this design leaves the device vulnerable to light exposure, which can result in photo-induced trapping phenomena and shifts in device characteristics such as Vth.

In contrast, the TG with light-blocking layer design incorporates a wider overlapping GC layer. This layer is engineered not only to provide electrical connectivity but also to integrate the light-blocking functionality. The GC layer is designed with a protective feature that extends over the active semiconductor region, effectively preventing ambient or incident light from reaching the underlying layers. This structural modification is crucial to reducing or eliminating photo-induced excitons and charge-trapping effects, which were a prominent issue in the TG-only design.

By adding this light-blocking feature to the GC layer, the modified design aims to improve the stability of the OTFT under illuminated conditions. The introduction of this layer ensures that the Vth remains stable, and hysteresis effects due to trapping are minimized. This design adjustment is particularly critical for applications where the OTFT must operate reliably in environments with varying light exposure, making the GC layer a central component in enhancing the device’s overall performance and robustness.

**Figure 8:**
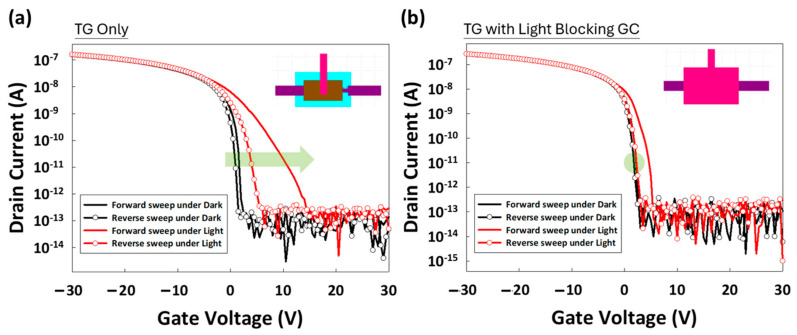
Comparison of the ID-VG of OTFT devices under two different designs: (**a**) TG-only and (**b**) TG with light-blocking layer. The measurements were taken both in the dark (black lines) and under illumination (red lines), with VG sweeping in forward and reverse directions. This comparison highlights the impact of the light-blocking layer on device performance, pink color represents the gate contact, which serves as the light-blocking layer, while brown color represents the TG.

In Figure 8, for the TG-only design (left), the ID-VG curve exhibits clear deviations under illumination compared to dark conditions. The forward and reverse sweeps under illumination (red lines) show significant hysteresis, as indicated by the wide separation between them. This hysteresis highlights the presence of photogenerated charge trapping and de-trapping processes triggered by light exposure. Additionally, a noticeable shift in Vth occurs, with the reverse sweep revealing charge accumulation and trapping, particularly under illuminated conditions. In contrast, the TG with light-blocking layer design (right) demonstrates improved stability under illumination. The forward and reverse sweeps under both dark and light conditions are more closely aligned, indicating reduced hysteresis. The light-blocking layer effectively prevents incident light from penetrating the active layer, thereby minimizing photo-induced trapping effects. The arrow in the figure highlights that the light-blocking layer contributes to a more stable and consistent ID-VG curve under illumination, resulting in less variation in Vth and a smaller hysteresis window compared to the TG-only design. This comparison clearly illustrates the effectiveness of the light-blocking layer in mitigating the adverse effects of illumination on OTFT performance, ultimately reducing trapped charges and improving the overall stability and reliability of the device in various lighting environments.

**Figure 9:**
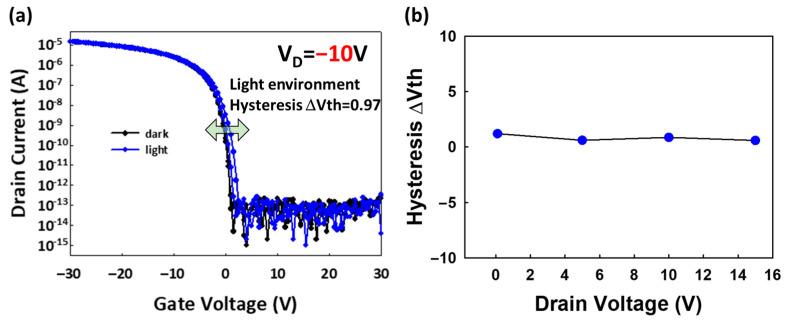
The ID-VG curve of the OTFT device under different VD conditions (−0.1 V, −5 V, −10 V, and −15 V), measured both in the dark (black curves) and under light exposure (blue curves).

Figure 9 demonstrates the impact of varying drain–source voltages (VD) on the electrical performance of the OTFT device under both dark and illuminated conditions. Since VD plays a crucial role in regulating the gamma voltage within the display circuitry, adjusting VD is necessary to achieve different grayscale for light-on images [32,33]. By monitoring the difference between the dark and light curves, we can observe a similar mechanism to that described earlier, indicating the generation of photo-induced charge carriers under illumination. This behavior is consistent with the previously discussed charge-trapping mechanism, where light exposure generates excitons that dissociate into free carriers within the OSC as trap states. These carriers mainly contribute to the energy band rather than the drain currents (ID). While VD increases, the electric field across the channel enhances the hole–carrier separation and movement from the semiconductor layer, but those photogenerated carriers do not increase correspondingly. This proves that the light-blocking layer design is effective in mitigating the impact of long-lived photocarriers in deep trap states. The results demonstrate improved electrical stability with reduced photocurrent generation, confirming that the light-blocking GC layer successfully minimizes the influence of persistent photogenerated carriers. This design effectively prevents these long-lived carriers from significantly altering the device’s behavior, enhancing the overall performance and reliability of the OTFT in illuminated environments.

**Figure 10:**
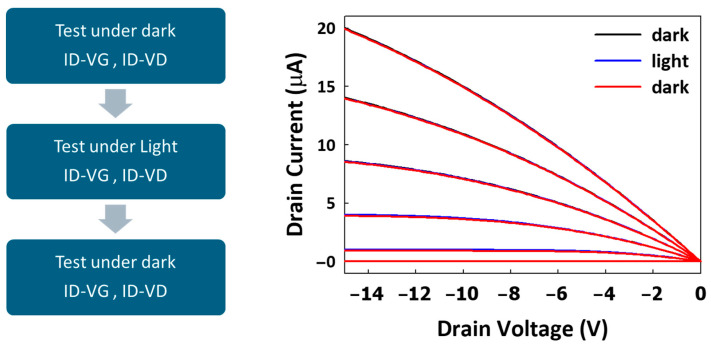
The testing procedure for the TG with light-blocking layer device, including the sequence of tests performed under different conditions.

To further verify the effectiveness of the light-blocking layer in suppressing photocurrent generation, an additional append test was conducted on the TG with light-blocking layer design. The test process flow, as shown in Figure 10, involved three phases: initial testing under dark conditions (ID-VG and ID-VD measurements), followed by testing under blue-light illumination, and concluding with a retest under dark conditions.

The ID-VD curves, presented in the right-hand graph of Figure 8, reveal the device behavior under these varying conditions. The results show that, under blue-light illumination, there are negligible increases in drain current (ID) across various VGs, as indicated by the red curves. Meanwhile, returning to dark conditions confirms that the light-induced effects are reversible, as the ID returns to baseline levels similar to the initial dark testing phase (blue curves). This indicates that the light-blocking layer (via GC) successfully minimizes photocurrent generation, enhancing the stability of the OTFT in illuminated environments.

## 4. Conclusions

In this work, we have explored the light stability of OTFTs integrated with MicroLED displays, with a particular focus on understanding the trapping mechanisms induced by light exposure. Our findings highlight that photo-induced trapping in OTFTs is a critical factor in influencing device performance, particularly in high-brightness operational environments. The introduction of a light-blocking layer within the OTFT structure proved to be an effective strategy in mitigating the long-lived photocarriers that contribute to threshold voltage shifts and hysteresis. By refining the OTFT design to include this protective layer, we have demonstrated improved device stability under varying light conditions, ensuring more reliable performance for MicroLED-driven applications. These results underscore the importance of considering light-induced effects in OTFT development and pave the way for further advancements in organic semiconductor technology tailored for display applications.

## Data Availability

The data presented in this study are available from the corresponding author upon reasonable request.
